# Distinct gut and oral microbial profiles differentiate patients with symmetric/asymmetric Parkinson’s disease

**DOI:** 10.3389/fnhum.2026.1776294

**Published:** 2026-03-17

**Authors:** Jia-Ying Jin, Dan Li, Shuang Qian, Fan Gao, Zong-Qin Li, Jin-Ru Zhang, Hong Jin, Fen Wang, Cheng-Jie Mao, Chun-Feng Liu, Xiao-Yu Cheng

**Affiliations:** 1 Department of Neurology and Clinical Research Center of Neurological Diseases, The Second Affiliated Hospital of Soochow University, Suzhou, China; 2 Department of Neurology, Suqian First Hospital, Suqian, China; 3 Jiangsu Key Laboratory of Drug Discovery and Translational Research for Brain Diseases, Institute of Neuroscience, Soochow University, Suzhou, China

**Keywords:** asymmetric, gut, microbiota, oral, Parkinson’s disease, symmetric

## Abstract

**Background:**

Parkinson’s disease (PD) presents heterogeneous motor patterns. Symmetric and asymmetric phenotypes potentially reflect distinct pathogenic origins as proposed by the Synuclein Origin and Connectome (SOC) model. However, differences in gut and oral microbiota between these PD subtypes remain unclear.

**Objective:**

To compare gut and oral microbiota characteristics in symmetric and asymmetric PD patients and explore correlations with clinical features.

**Methods:**

Thirty symmetric and twenty-three asymmetric PD patients were enrolled. Fecal and salivary microbiota were analyzed using 16S rRNA sequencing, and clinical features were evaluated using standard motor and non-motor scales.

**Results:**

The symmetric group showed higher H–Y stage and scores of MDS-UPDRS II, DSFS, MMSE, and MoCA than the asymmetric group (all *p* < 0.05). Gut and oral microbiota structures differed significantly, with higher gut microbial *α*-diversity in the symmetric group. Desulfobacterota and related taxa were enriched in symmetric PD and correlated positively with GCSI scores, while butyrate-producing bacteria predominated in asymmetric PD. Predicted metabolic analyses indicated enrichment of six pathways in asymmetric gut microbiota and nine pathways enriched in symmetric oral microbiota.

**Conclusion:**

Symmetric and asymmetric PD are associated with distinct clinical and gut microbiota features, including predicted functional profiles. This aligns with the SOC model of divergent disease origins and mechanisms and points to the microbiota as a candidate factor in PD heterogeneity, offering new testable hypotheses for future research.

## 1 Introduction

Parkinson’s disease (PD), recognized as the second most prevalent neurodegenerative disorder worldwide, is pathologically defined by the abnormal accumulation of *α*-synuclein aggregates (Magalhães and Lashuel, 2022). Clinically, it manifests through a spectrum of motor and non-motor symptoms, with motor features including resting tremor, bradykinesia, and rigidity (Leite Silva et al., 2023). Notably, significant interpatient variability exists, with some PD patients present with symmetric motor symptoms, while others exhibit asymmetry. This striking clinical heterogeneity, particularly in movement asymmetry patterns, has emerged as a critical focus of contemporary neurological research (Djaldetti et al., 2006; Van Den Berge et al., 2019; Metcalfe-Roach et al., 2024; Woo et al., 2024). According to previous reports, PD patients with symmetric symptom have faster progression, higher risk of balance disorders, more restrictions on daily activities, higher risk of restless leg syndrome, poorer mental state, and higher risk of cognitive impairment and dementia (Lichter et al., 2018). The heterogeneity of clinical manifestations of PD may originate from different initial accumulation sites of pathological *α* - synuclein, and the hypothesis of α-synuclein Origin and Connectome Model (SOC Model) is proposed (Borghammer, 2021). The hypothesis suggests that pathological changes can originate from the central nervous system (CNS), and these patients exhibiting predominantly asymmetric motor symptoms. Pathological changes can also originate from the periphery, such as the enteric nervous system (ENS) (Pasricha et al., 2024), and these patients present with symmetric symptoms (Breid et al., 2016; Kim et al., 2019). At present, clinical studies on PD patients with symmetric/asymmetric phenotype mainly focus on clinical manifestations, progression, and imaging features. A study in 2024 showed that there were differences in gut microbiota between PD patients and healthy individuals, and the enrichment trend was more significant in symmetric PD, suggesting that abnormal microbiota may play a role in the pathogenesis of symmetric/asymmetric PD patients (Metcalfe-Roach et al., 2024). However, there is currently a lack of comprehensive analysis on the characteristics of gut microbiota in patients between symmetric and asymmetric PD. In addition, the gut and oral microbiota represent the different parts in the digestive tract, showing the correlations with diseases, respectively (Berthouzoz et al., 2023). Therefore, this study included PD patients with two different movement distribution patterns (symmetric/asymmetric), analyzed their clinical characteristics, and performed 16S rRNA to detect and study the microbiota characteristics in their feces and saliva. The aim is to elucidate the possible role of gut microbiota in the disease origin and pathogenesis of PD patients with symmetric or asymmetric presentation, and provide important information for the study of PD pathogenesis.

## 2 Materials and methods

### 2.1 Patient recruitment

In the study, we recruited 53 patients with idiopathic PD from neurology department of the Second Affiliated Hospital of Soochow University between November 2022 to March 2024. All PD patients were diagnosed based on the International Parkinson and Movement Disorder Society’s (MDS) diagnostic criteria (2015 edition). The exclusion criteria were as follows: (1) Serious cardiovascular and cerebrovascular diseases, mental illnesses, digestive system diseases, infections and other debilitating diseases; (2) Patients who have taken drugs or antibiotics that affect gastrointestinal function in the past 3 months. (3) patients with active periodontal diseases, such as gingivitis, periodontitis, dental surgery within the past 3 months, or significant dental caries during the screening phase. (4) Patients with self-reported extreme dietary patterns, specifically vegan diet, high-fat or high glucose diet, obesity, or malnutrition. This study has been were approved by the Ethics Committee of the Second Affiliated Hospital of Soochow University (Approval No: JD-LK-2018-061-03), and has obtained informed consent from all research subjects.

### 2.2 Clinical data collection

General clinical data of patients are collected, including age, gender, height, weight, body mass index (BMI), and levodopa equivalent daily dose (LEDD). The motor and non motor symptoms of PD were assessed using scales, which were evaluated by professional neurologists. The H-Y Stage, Movement Disorders Unified Parkinson’s Disease Rating Scale III (MDS-UPDRS III) were performed only in *on*-state. Drooling Severity and Frequency Scale (DSFS), Drooling Rating Scale (DRS), Sialorrhea Clinical Scale for Parkinson’s Disease (SCS-PD), and Eating Assessment Questionnaire (Assessment Tool-10, EAT-10), Gastroparesis Cardinal Symptom Index (GCSI), Rome IV Constipation (ROME) (IV) Standard judgment were performed to judge the digestive symptoms. Other non motor symptoms were evaluated by Mini-Mental State Examination (MMSE), Montreal Cognitive Assessment (MoCA), Hamilton Anxiety Rating Scale (HAMA), Hamilton Depression Rating Scale-24 (HAMD-24), Epworth Sleepiness Scale (ESS), Rapid Eye Movement Sleep Behavior Disorder Questionnaire Hong Kong (RBD-HK), Rapid Eye Movement Sleep Behavior Disorder screening questionnaire (RBDSQ), Parkinson’s Disease Sleep Scale-2 (PDSS-2), Scales for outcomes in Parkinson’s Disease-Autonomic (SCOPA-AUT), Fatigue Severity Scale (FSS), and Non motor Symptoms Questionnaire (NMSQ).

### 2.3 Define motor symmetric and asymmetric groups

The distinction between the motor symmetric group and motor asymmetric group is determined by calculating the absolute difference between the average scores of the left and right sides for items 3.3–3.8 and 3.15–3.17 in the baseline MDS-UPDRS Part III. Patients are classified into the motor asymmetric group if the difference exceeds the median value, and into the motor symmetric group otherwise.

### 2.4 Sample collection

Fecal and salivary samples from participants were immediately refrigerated after collection. Sampling tubes were transferred to a − 80 °C ultra-low temperature freezer within 24 h. Upon completion of sample collection, all specimens were transported to the laboratory in the same batch, packed with sufficient dry ice, and analyzed simultaneously to minimize repeated freeze–thaw cycles.

### 2.5 Data processing and analysis

Genomic DNA was extracted using the MagPure Soil DNA LQ Kit (Magan, China), which has been validated for fecal and salivary samples (Jin et al., 2025; Mei et al., 2024). The bacterial *16S rRNA* gene was amplified using extracted DNA as the template. PCR amplicons underwent paired-end sequencing (2 × 250 bp) on the Illumina NovaSeq 6,000 platform. Taxonomic annotation was performed using the q2-feature-classifier module with the SILVA 138 database. Alpha diversity was quantified via the Chao1 index and Phylogenetic Diversity (PD) whole tree index with two-tailed Student’s *t*-test (*p* < 0.05). Differential species abundance profiles were analyzed using Linear discriminant analysis Effect Size (LEfSe). Differential taxa were identified using LEfSe with a Kruskal–Wallis test (*p* < 0.05) and a linear discriminant analysis (LDA) score threshold of log10 > 2.0. In silico functional prediction of microbial communities was conducted via Phylogenetic Investigation of Communities by Reconstruction of Unobserved States (PICRUSt), referencing the Kyoto Encyclopedia of Genes and Genomes (KEGG) Orthology (KO) database. Spearman’s rank correlation coefficient was employed to construct association networks, and key functional modules were analyzed using the Python SciPy toolkit.

### 2.6 Statistical analysis

Data analysis was conducted using IBM SPSS Statistics 26.0 (IBM Corporation, Armonk, NY, USA). Shapiro Wilk method was applied to verify normality and Levene method to evaluate homogeneity of variance. Continuous variable representation adopts a bimodal approach, when it conforms to a normal distribution, it is presented as mean ± standard deviation and independent sample t-test is applied. Non normal data are presented as median (IQR) and Mann Whitney U test is performed. The categorical variables are characterized by frequency distribution, and the differences between groups are achieved through Wilcoxon rank sum test.

## 3 Results

### 3.1 Demographic and clinical characteristics

A total of 70 PD patients fulfilling the defined inclusion/exclusion criteria were initially enrolled in this study and clinical data were collected. 15 participants failed to provide biological specimens, and 53 patients were ultimately enrolled. Including 32 males (60.3%) and 21 females (39.6%). According to the MDS-UPDRS III assessment, which evaluates the symmetric of motor symptoms on both sides, 53 patients were divided into two groups: 30 patients in the motor symmetric group (56.6%) and 23 patients in the motor asymmetric group (43.4%). There were no significant differences in the gender, age, Body Mass Index (BMI), course of disease, levodopa equivalent dose (LEDD), MDS-UPDRS I and III parts (all *p* > 0.05). Compared with the motor asymmetric group, the H-Y staging of the motor symmetric group was higher, as well as the scores of MDS-UPDRS II scores, Drooling Severity and Frequency Scale (DSFS), Mini-Mental State Examination (MMSE), Montreal Cognitive Assessment (MoCA) (all *p* < 0.05, Table 1).

**Table 1:** Demographic and clinical characteristics of motor symmetric/asymmetric PD patients.

Characteristics	PD-SYM	PD-ASYM	*p*
Number of patients	30	23	/
Male (%)	18 (60.0)	14 (60.9)	0.949
Age (years)	65.2 ± 8.1	65.6 ± 8.5	0.863
BMI (kg/m^2^)	23.6 ± 3.0	24.3 ± 1.9	0.324
Duration of disease (month)	54.0 (26.0, 89.0)	57.0 (16.0, 97.0)	0.907
H-Y stage	2.0 (2.0, 2.5)	2.0 (1.5, 2.0)	0.041*
LEDD (mg)	450.0 (314.1, 728.1)	375.0 (100.0, 537.5)	0.201
Levodopa (%)	27 (90.0)	19 (82.6)	0.705
DR agonist (%)	21 (70.0)	11 (47.8)	0.102
MAOB inhibitor (%)	15 (50.0)	6 (26.1)	0.078
COMT inhibitor (%)	8 (26.7)	6 (26.1)	0.962
Amantadine (%)	7 (23.3)	3 (13.0)	0.552
MDS-UPDRS I	7.0 (3.8, 13.3)	5.0 (3.0, 11.3)	0.217
MDS-UPDRS II	9.0 (5.0, 14.0)	4.0 (3.0, 8.0)	0.039*
MDS-UPDRS III	33.3 ± 16.1	30.9 ± 15.9	0.586
DSFS	4.0 (2.0, 7.0)	2.0 (2.0, 4.0)	0.023*
SCS-PD	4.0 (0.5, 8.5)	1.0 (0.0, 5.0)	0.067
DRS	1.0 (0.0, 5.0)	0.0 (0.0, 4.0)	0.136
EAT-10	0.0 (0.0, 2.5)	0.0 (0.0, 2.0)	0.721
GCSI	0.0 (0.0, 2.0)	0.0 (0.0, 3.0)	0.868
ROME IV (%)	15 (50.0)	11 (47.8)	0.875
CSS	7.00 (0.00, 11.50)	8.00 (1.00, 10.00)	0.963
Laxative use (%)	15 (50.0)	8 (34.8)	0.268
MMSE	29.0 (26.0, 30.0)	25.0 (23.0, 29.0)	0.044*
MoCA	23.1 ± 4.3	20.0 ± 4.9	0.018*
HAMD-24	8.5 (2.0, 14.0)	5.0 (1.8, 17.5)	0.759
HAMA	7.5 (2.0, 12.3)	6.5 (1.0, 13.5)	0.831
ESS	5.0 (2.0, 9.0)	3.0 (0.0, 10.5)	0.640
RBD-HK	8.0 (3.3, 20.0)	9.0 (4.8, 27.5)	0.341
RBDSQ	0.5 (0.0, 4.0)	2.0 (0.0, 4.3)	0.503
PDSS-2	8.0 (4.0, 15.8)	9.0 (4.0, 13.5)	0.753
NMSQ	8.0 (4.5, 10.5)	4.5 (3.0, 9.0)	0.249
SCOPA-AUT	6.0 (3.5, 12.5)	6.0 (1.5, 12.0)	0.601
FSS	34.0 (22.0, 45.8)	34.0 (9.0, 46.8)	0.421

PD-SYM, symmetric PD; PD-ASYM, asymmetric-PD; BMI, Body mass index; H-Y stage, Hoehn-Yahr stage; LEDD, Levodopa Equivalent Daily Dose; DR, dopamine receptor; MAOB, monoamine oxidase B; COMT, catechol- O- methyltransferase; MDS-UPDRS, Movement Disorders-Unified Parkinson’s Disease Rating Scale; DSFS, Drooling Severity and Frequency Scale; DRS, Drooling Rating Scale; SCS-PD, Sialorrhea Clinical Scale for Parkinson’s disease; EAT-10, Eating Assessment Tool-10; GCSI, Gastroparesis Cardinal Symptom Index; CSS, Constipation Scoring System; MMSE, Mini-Mental State Examination; MoCA, Montreal Cognitive Assessment; HAMD-24, Hamilton Depression Scale-24; HAMA, Hamilton Rating Scale for Anxiety; ESS, Epworth Sleepiness Scale; RBD-HK, Rapid eye movement Sleep Behavior Disorder Questionnaire Hongkong; RBDSQ, Rapid eye movement Sleep Behavior Disorder screening questionnaire; PDSS-2, Parkinson’s Disease Sleep Scale-2; NMSQ, Non-motor Symptoms Questionnaire; SCOPA-AUT, Scales for Outcomes in Parkinson’s Disease-Autonomic; FSS, Fatigue Severity Scale. “*” represents *p* < 0.05.

### 3.2 Analysis of gut and oral microbiota structure and diversity in patients with motor symmetric/asymmetric PD

**Figure 1:**
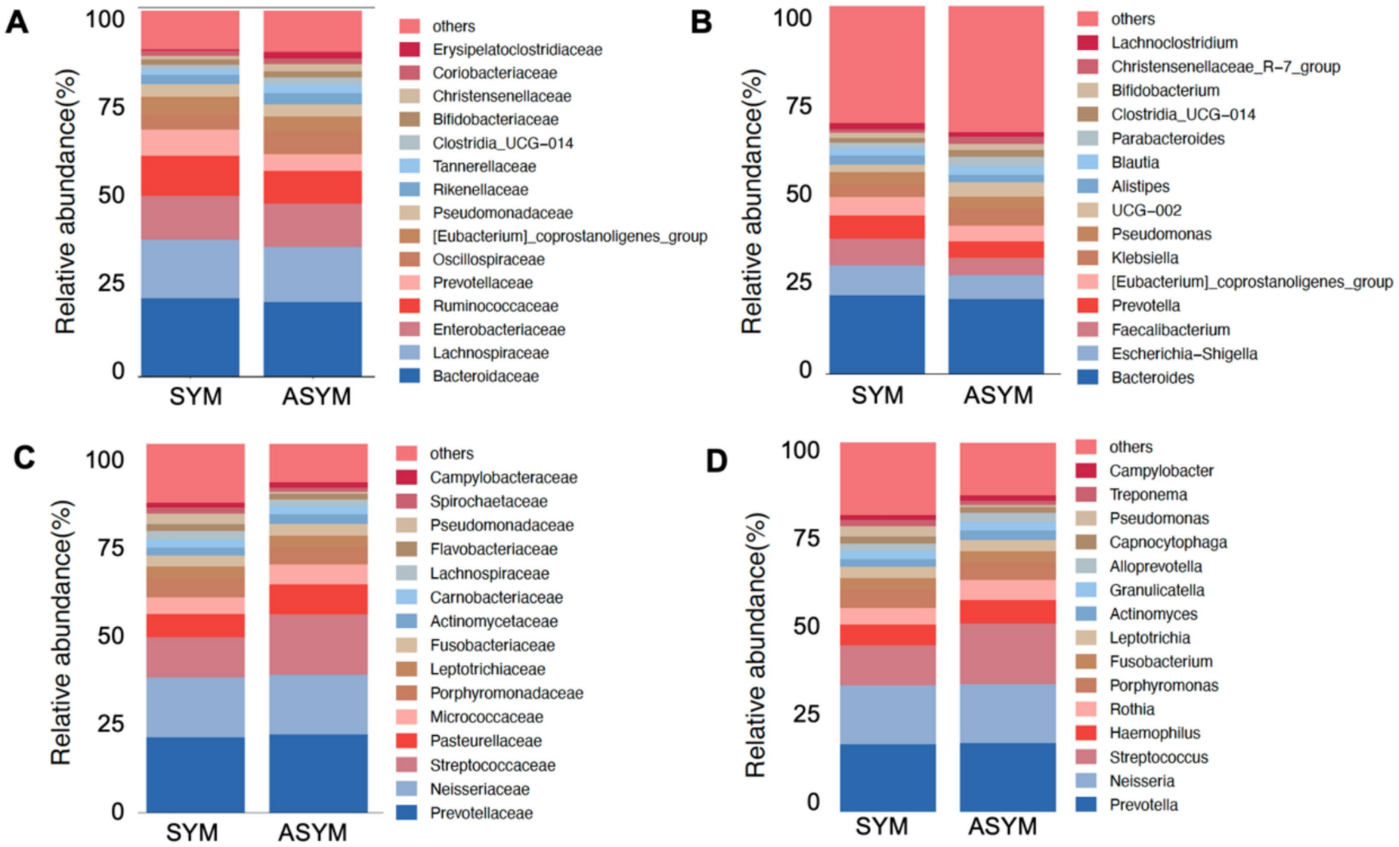
Gut and oral microbiota distinct structure in symmetric and asymmetric PD patients. **(A)** There are differences in the composition of gut microbiota between symmetric and asymmetric PD patients at the family level. **(B)** There are differences in the composition of gut microbiota at the genus level between symmetric and asymmetric PD patients. **(C)** There are differences in the composition of oral microbiota between symmetric and asymmetric PD patients at the family level. **(D)** There are differences in the composition of oral microbiota between symmetric and asymmetric PD patients at the genus level.

Based on the gut and oral species annotation results, the top 15 highest abundance species at the family and genus levels for motor symmetric/asymmetric subtypes were selected, and the variation of microbial structure between these two groups was observed (Figures 1A–D).

**Figure 2:**
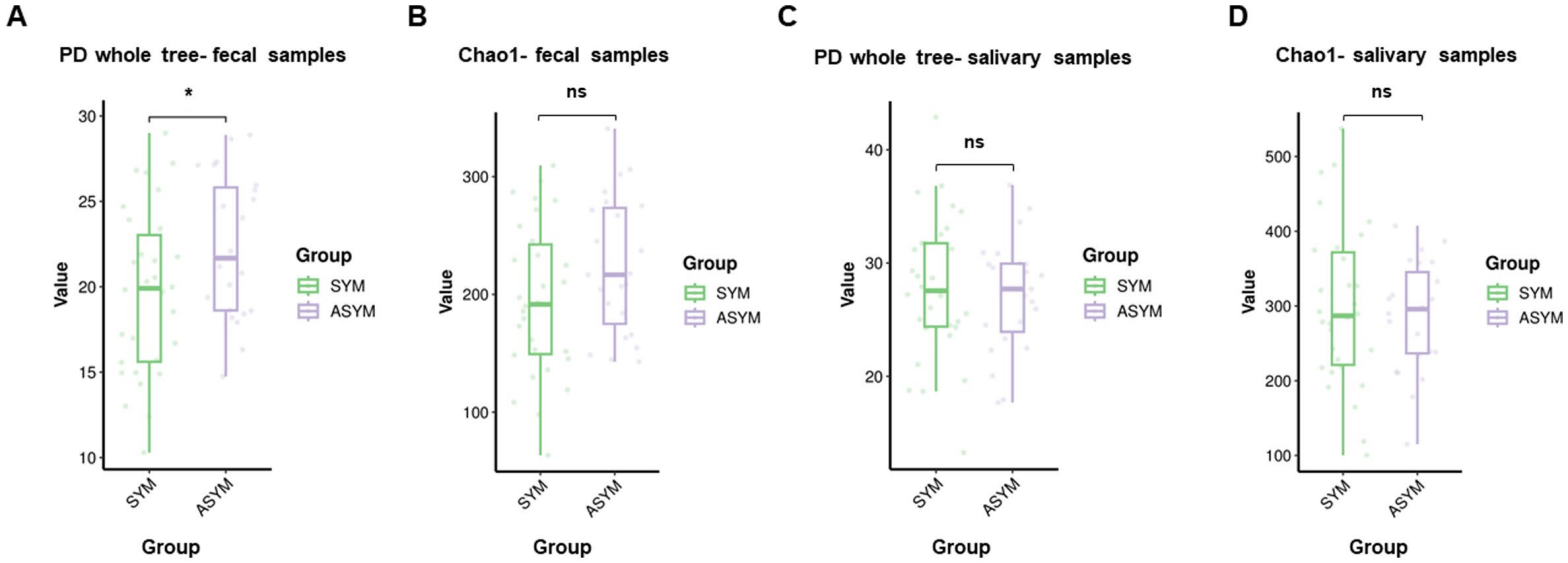
Alpha diversity of gut and oral microbiota in symmetric and asymmetric PD patients. Alpha diversity indices of the gut microbiota in the fecal samples: **(A)** PD whole tree index, **(B)** Chao1 index; alpha diversity indices of the oral microbiota in the salivary samples: **(C)** PD whole tree index, **(D)** Chao1 index. Data are presented as box plots showing median and interquartile range. Statistical comparisons between symmetric and asymmetric PD groups were performed using two-tailed Student’s *t*-test. “*” represents *p* < 0.05 and “ns” indicates no statistically significant difference.

The overall alpha diversity was evaluated by PD whole tree index and chao1 index. We found the diversity of gut microbiota was higher in the motor asymmetric group compared with the motor symmetric group, according to the PD whole tree index (*p* < 0.05). There was no significant difference in the diversity of oral microbiota between the two groups (Figures 2A–D).

### 3.3 Distinct microbiota in fecal and saliva between motor symmetric/asymmetric PD at different taxonomic levels

**Figure 3:**
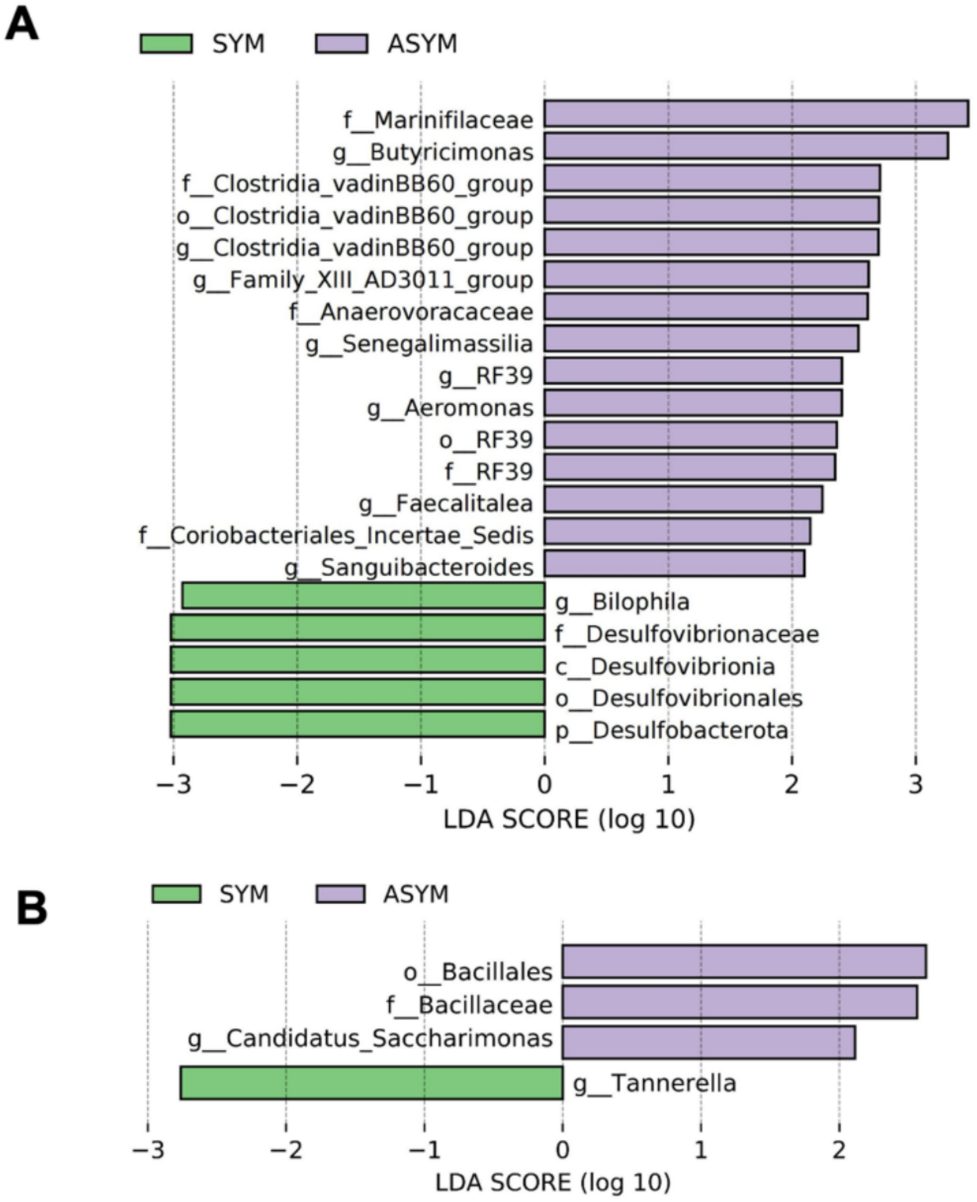
LEfSe analysis identifying differentially abundant taxa between symmetric and asymmetric PD patients. Linear discriminant analysis effect size (LEfSe) was performed to identify taxa with significant differences between groups, using a Kruskal–Wallis test with *p* < 0.05 and an LDA score threshold of log_10_ > 2.0. Positive LDA scores (purple) represent taxa enriched in asymmetric PD patients (ASYM), whereas negative LDA scores (green) indicate taxa enriched in symmetric PD patients (SYM) across fecal **(A)** and salivary **(B)** samples.

The LEfSe analysis integrated Kruskal-Wallis test, Wilcoxon test, and Linear Discriminant Analysis (LDA) to indicate differential microbial taxa between these two groups. In gut microbiota, the motor symmetric group exhibited enrichment in phylum Desulfobacterota, as well as its subordinate taxa including class Desulfovibrionia, order Desulfovibrionales, and family Desulfovibrionaceae compared to the motor asymmetric group. The genus *Bilophila* also exhibited higher abundance in the symmetric group. Conversely, the motor asymmetric group showed significant enrichment of the class Clostridia, order RF39, Family_XIII, family Anaerovoracaceae, and genus *Faecalitalea* within the phylum Firmicutes, and the family Marinifilaceae and genus *Butyricimonas* within the phylum Bacteroidetes, as well as the order Coriobacteriales within the phylum Actinobacteria. Additionally, the genera *Aeromonas* and *Sanguibacteroides* also displayed asymmetric group-specific enrichment. In the oral microbiota, the genus *Tannerella* within the phylum Bacteroidetes was enriched in the motor symmetric group. In contrast, the order Bacillales and its subordinate family Bacillaceae within the phylum Firmicutes, as well as the genus *Candidatus Saccharimonas* were enriched in the motor asymmetric group. The LDA score quantifies the effect of statistically significant intergroup differences, as described in Figure 3.

### 3.4 Distinct metabolic changes associate between motor symmetric/asymmetric PD

**Figure 4:**
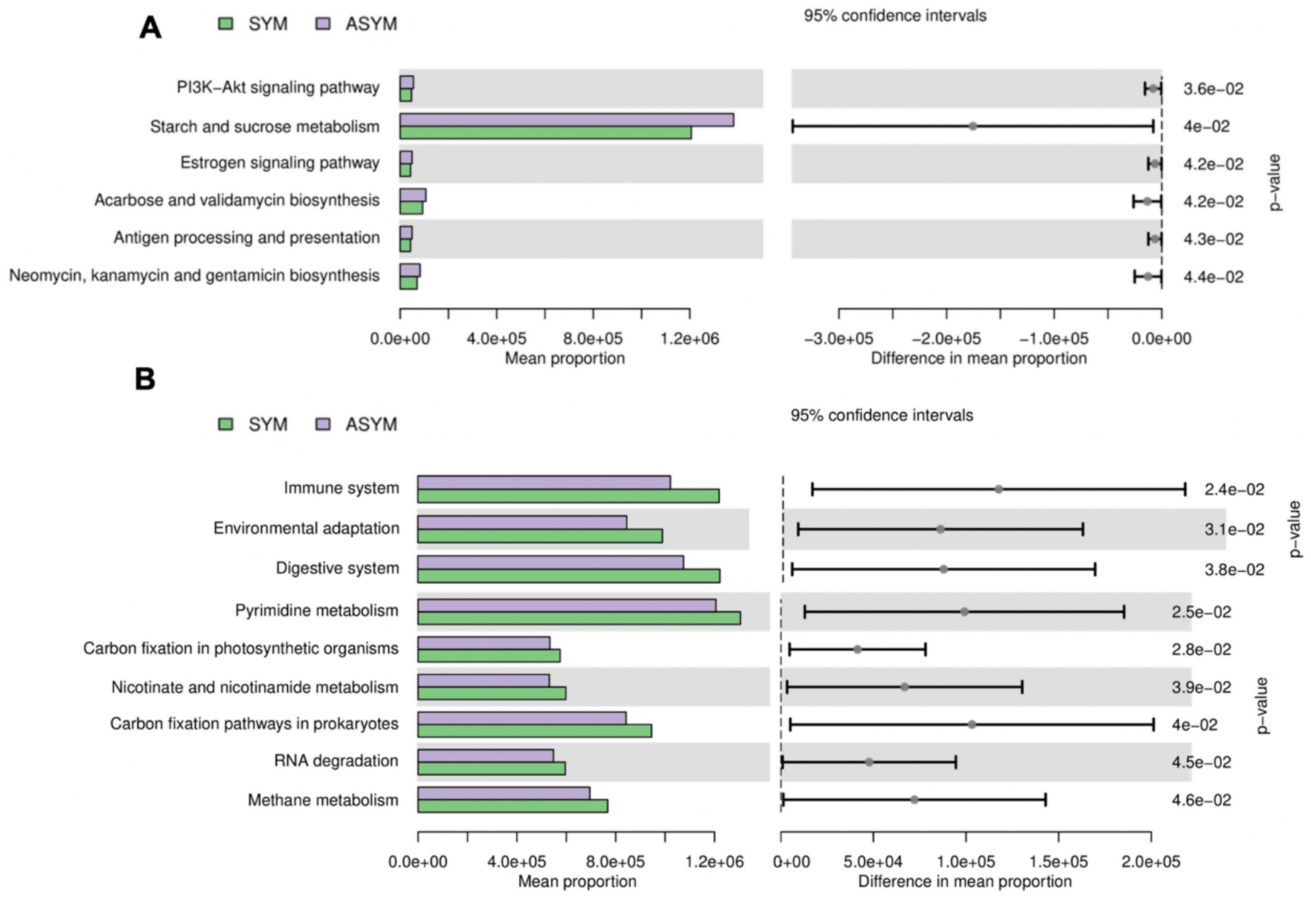
Predicted differential metabolic pathways (KEGG level 3) between symmetric and asymmetric PD patients. Functional prediction was performed using PICRUSt based on 16S rRNA gene sequencing data, and statistical analysis was conducted using STAMP with a Welch’s *t*-test (*p* < 0.05). Green bars represent pathways enriched in symmetric PD patients (SYM), whereas purple bars represent pathways enriched in asymmetric PD patients (ASYM), displayed with 95% confidence intervals (CI) for the difference in mean proportions across fecal **(A)** and salivary **(B)** samples.

PICRUSt was employed to predict the functional profiles of the microbiota in these two groups. Based on predicted microbial community functional potential, we identified KOs demonstrating significant differential abundance (FDR adjusted *p* < 0.05) between motor symmetric/asymmetric PD from fecal and salivary samples. Analysis of predicted pathway abundances revealed six and nine differentially abundant pathways in the gut and oral microbiota, respectively. In the gut microbiota, the asymmetric group showed a predicted enrichment in pathways related to PI3K-Akt signaling, starch and sucrose metabolism, estrogen signaling pathway, acarbose and validamycin biosynthesis, antigen processing and presentation, and antibiotic biosynthesis (Figure 4A). In the oral microbiota, the symmetric group had more abundant pathways related to the immune system, environment, digestion, pyrimidine metabolism, carbon fixation, niacin and nicotinamide metabolism, RNA degradation, and methane metabolism (Figure 4B).

### 3.5 Correlation analysis between microbiota and clinical features

**Figure 5:**
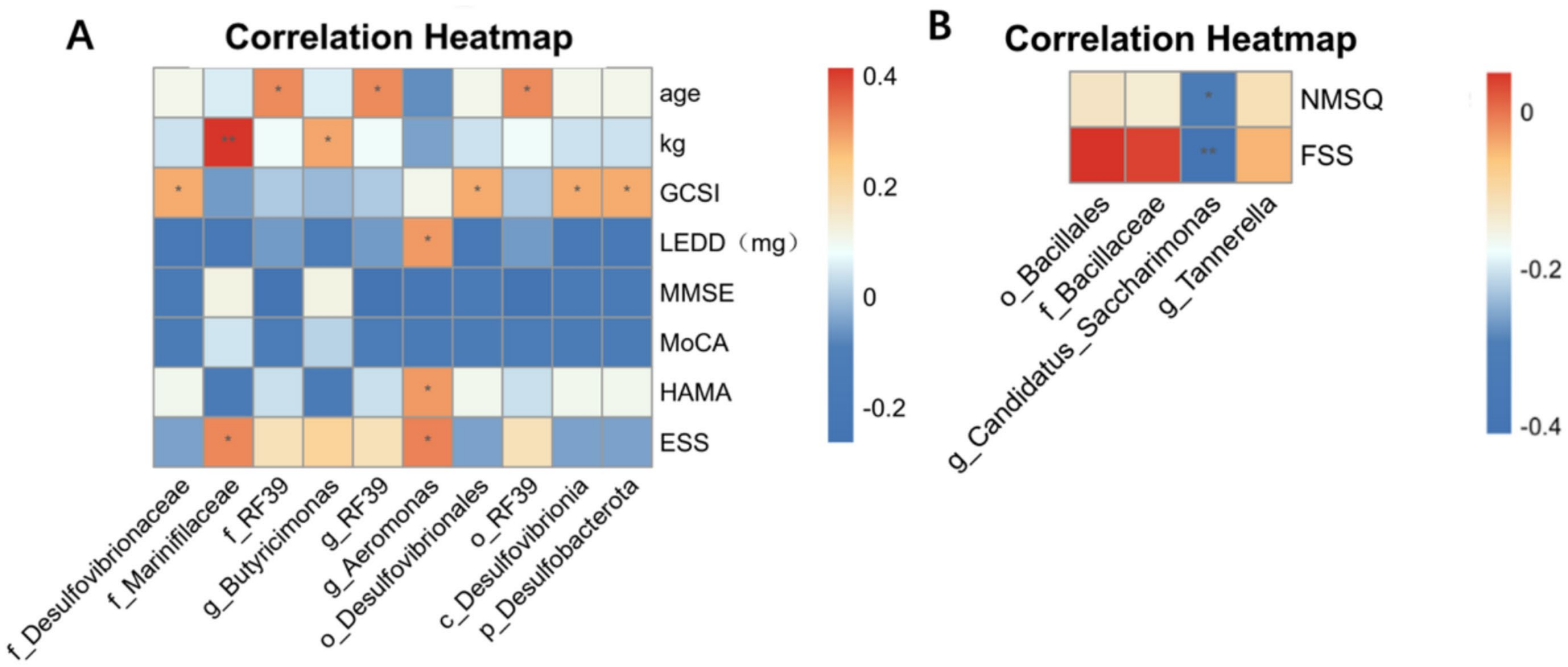
Spearman correlation analysis between differentially abundant taxa and clinical manifestations in PD patients. The analysis was performed based on 16S rRNA gene sequencing data and clinical metadata, calculated using Spearman’s rank correlation coefficient. **(A)** Correlations between clinical features and differentially abundant taxa in fecal samples. **(B)** Correlations between clinical features and differentially abundant taxa in salivary samples. The color scale indicates the strength of the correlation, where red represents positive correlations and blue represents negative correlations. Asterisks denote statistical significance. “*” represents *p* < 0.05 and “**” represents *p* < 0.01.

Next, we selected the microbiota with significant differences between groups selected from LEfSEs analyze and revealed the correlation between their abundance and clinical symptoms of PD. In the gut microbiota, the enrichment of phylum Desulfobacterota and its subordinate species in the symmetric group of motor symptoms was positively correlated with GCSI (*r* = 0.28, *p* < 0.05). The enrichment of Marinifilaceae in the motor asymmetric group was positively correlated with body weight (*r* = 0.41, *p* < 0.01) and ESS (*r* = 0.32, *p* < 0.05). The enrichment of RF39 was positively correlated with age (*r* = 0.32, *p* < 0.05). The enrichment of *Butyricimonas* was positively correlated with body weight (*r* = 0.28, *p* < 0.05). The enrichment of *Aeromonas* was positively correlated with LEDD (*r* = 0.30, *p* < 0.05), HAMA (*r* = 0.30, *p* < 0.05), and ESS (*r* = 0.33, *p* < 0.05). In oral microbiota, the enrichment of *Candidatus* in the motor asymmetric group was negatively correlated with NMSQ (*r* = −0.33, *p* < 0.05) and FSS (*r* = −0.48, *p* < 0.01) (Figure 5).

## 4 Discussion

We investigated the difference in clinical manifestations and microbiota between PD individuals with symmetric or asymmetric presentation. We consequently attempted to explain the potential association of microbiota with clinical manifestations and motor subtypes. Providing H-Y stage clinical evidence to support for SOC model theory.

Our study showed a significant difference in clinical feature between the motor symmetric and asymmetric groups. A higher H-Y stage was associated with the symmetric group. In addition, compared with asymmetric individuals, the MDS-UPDRS II, DSFS, MMSE, and MoCA scores were also higher than the motor symmetric ones, indicating that motor symmetric patients had more severe daily activity problems and more severe salivation troubles, while a better cognitive function. Previous studies have shown that patients in the motor symmetric group have lower quality of daily life (Marinus and van Hilten, 2015), which is consistent with our findings. There is currently no research on the salivation between these two groups. The SOC Model hypothesis suggests that the pathology of PD do not always originate from the brain. Pathological changes originating from the cerebral hemisphere result in asymmetric motor symptoms on the onset side, whereas those originating from peripheral nervous system subsequently ascend to the brain via the vagal nerves, which leads to symmetric motor symptoms, reflecting a wider involvement of brainstem and bilateral cerebral hemisphere. These patients imply a broader burden of *α*-synuclein, including regions controlling autonomic nervous system, whose dysfunction can disrupt coordinated saliva secretion and swallowing mechanisms, potentially resulting in excessive salivation symptoms in patients. However, there was no significant difference assessment in swallowing, gastroparesis, constipation, fatigue, and sleep between these two groups. Perhaps due to the complex causes of non-motor symptoms, which involve the synergistic control between peripheral and brain. For example, gastrointestinal function being regulated not only by the CNS but also by the local ENS. In addition, the number of participants enrolled in our study is relatively limited, and future larger sample studies might be necessary. Furthermore, previous study has reported that the motor symmetric PD patients have more severe cognitive impairment. On the contrary, our data showed higher MMSE and MoCA scores in the symmetric group. However, this observation should be interpreted with caution. It may reflect differences in enrolled populations, disease duration, or other unmeasured confounders. It does not necessarily imply that motor symmetric is intrinsically linked to preserved cognition in PD. Longitudinal studies tracking cognitive trajectories alongside motor symmetric are needed to clarify this relationship (Uc et al., 2009).

The 16S rRNA sequencing indicated the composition difference of gut and oral microbiota between motor symmetric and asymmetric PD patients. Our result showed the overall structure and diversity differentia of microbiota in these two groups, and gut microbiota displayed more significant distinction. In the gut of motor symmetric group, the abundance of Phylum Desulfobacterota and the subordinate taxonomic units increased. *Desulfovibrio* is a typical sulfate-reducing bacterium (SRB), which participates in the oxidation–reduction reactions of sulfate/thiosulfate. Specifically, *Desulfovibrio* consumes sulfate and reduces it to hydrogen sulfide (H ₂ S) or sulfide (S ^2 −^), thereby directly reducing sulfate concentration. In recent years, attention regarding sulfur metabolism has grown in PD research field. Copper sulfate has been reported to prevent tyrosine hydroxylase (TH) activity reduced and motor deficits in a PD model (Alcaraz-Zubeldia et al., 2009), indicating the protective effect of sulfates in PD. Moreover, the high level of *Desulfovibrio* are positively correlated with the motor symptoms and cognitive function in PD (Murros et al., 2021). Animal experiments also have shown that *Desulfovibrio* can promote the aggregation of *α*-synuclein and spread them through the brain-gut axis (Huynh et al., 2023). Our data further suggested that *Desulfovibrio* is PD symmetric phenotype associated, which may explain the more severe clinical feature and pathology in the motor symmetric group. This is consistent with a reported viewpoint, which stated that most PD associated microbial trends were stronger in those with symmetric motor symptoms (Metcalfe-Roach et al., 2024). *Bilophila* was also significantly enriched in the motor symmetric group. Previous studies reported that it participated in intestinal inflammation and cognitive dysfunction (Shi et al., 2022; Du et al., 2020). Experimental and clinical study data both suggest that intestinal inflammation is a driver of PD (Li et al., 2021; Houser and Tansey, 2017), and the abundance of *Bilophila* in symmetric PD may indicate more severe intestinal inflammation and pathogenic factors.

The relevance analysis of microbiota species with clinical symptoms also showed that *Bilophila*, phylum Desulfobacterota and its subordinate taxonomic units were negatively correlated with MMSE and MoCA scores. Furthermore, *Desulfovibrio* is positively correlated with GCSI and UPDRS-I. Although the correlation is only significant between the *Desulfovibrio* and GCSI, it still suggests that these species are associated with the exacerbation of cognitive function and non motor symptoms. The predicted enrichment of the PI3K-Akt pathway suggests that the higher abundance of potentially pathogenic taxa in the symmetric group may contribute to the clinical severity of PD, possibly through mechanisms involving intestinal metabolism and inflammation.

In the motor asymmetric group, Clostridia, RF39, Family_XIII, Anaerovoracaceae, Faecalitalea, Marinifelaceae and Coriobacterales increased, which belong to the phylum Firmicutes. In addition, the enrichment of genus *Butyricimonas* belong to the phylum Bacteroidetes. *Clostridium*, Family_XIII, Anaerovoracaceae and Faecalitalea were thought to maintain the integrity of the intestinal barrier by producing short chain fatty acids (SCFAs), such as butyric acid (Zhu et al., 2022; Yang et al., 2025; Shah et al., 2024). Among them, Family_XIII also participates in carbohydrate fermentation in the intestine, which may participate in maintaining intestinal homeostasis by regulating intestinal pH and energy metabolism. Family XIII can also product the indole metabolites (such as skatole and indole), which contributes in systemic inflammation and metabolic regulation through the gut-brain axis or gut-liver axis (Shi et al., 2020). RF39 belongs to an unclassified family or genus unit under the phylum Firmicutes, and their taxonomic status requires further validation through genome sequencing. The other abundance taxa is the genus *Butyricimonas,* functionally characterized by its core metabolite, butyrate. Butyrate serves as the main energy source for intestinal epithelial cells, which can enhance intestinal barrier function, inhibit the release of pro-inflammatory factors (such as TNF - *α*, IL-6), regulate immune responses, and delay abnormal aggregation of α-synuclein (Salvi and Cowles, 2021; Hirayama et al., 2023). The reduction of butyrate-producing bacteria was associated with impaired colonic health in other researches, and its lower abundance in PD may similarly reflect a dysbiotic state (Nuzum et al., 2023; Stoeva et al., 2021). Therefore, *Butyricimonas* is generally considered a healthy bacterial community, and the abundance of *Butyricimonas* in PD patients’ feces was thought to be lower than that of healthy individuals (Stoeva et al., 2021). Moreover, low levels of *Butyricimonas* usually indicate more severe non motor symptoms (Nuzum et al., 2023). The correlation analysis with clinical symptoms showed that the abundance of *Butyricimonas* was negatively associated with the scores of SCOPA-AUT, PDSS-2, HAHM, HAMA, LEDD, MDS-UPDRS I/ II/III, EAT-10. Although the correlation does not reach statistical significance, it indicates these microbiota might mitigate motor and non-motor symptom burden. Future research needs to enroll a larger sample to validate our hypothesis. Coriobacteriales is associated with antioxidative stress and intestinal inflammation (Huang et al., 2019; Prasad and Bondy, 2018). *Senegalimassilia* participates in the breakdown of dietary fiber and the synthesis of SCFA. *Aeromonas* belongs to the Vibrionaceae family and is a conditionally pathogenic bacterium associated with intestinal inflammation and tissue invasion (Pessoa et al., 2022). The role of *Sanguibacteroides* in the intestine has not been extensively studied. The above microbiota show a trend of mitigating PD motor and nonmotor symptoms, but there are currently no reports related to PD. Its role in PD needs to be further elucidated through the combination of metagenomics, metabolomics, and animal models in the future.

Research on oral microbiota’s role in PD remains limited, however, the oral cavity is a critical upper gastrointestinal compartment and establishes direct neuroanatomical connections (trigeminal, olfactory, and facial nerves) that may facilitate brain-targeted transmission via neural pathways. A study shows that a large number of *Porphyromonas gingivalis* DNA fragments have been detected in the brains of Alzheimer’s disease patients (Dominy et al., 2019), indicating a close association between oral bacteria and degenerative diseases of the CNS. Our results showed that Tannerella was symmetric associated. *Tannerella* belongs to the Bacteroidetes phylum, which has been shown to be related to oral diseases such as periodontal disease. As a conditional pathogen, it causes disease by affecting glucose metabolism, promoting inflammation, and affecting mucosal integrity (Xia et al., 2025). Correlational analyses revealed that increased microbial abundance exhibited associations with elevated LEDD and higher RBD-HK severity scores, indicating potential involvement in both dopaminergic therapy escalation and REM sleep behavior disorder progression. In the motor asymmetric group, the abundance of Bacillales and its subordinate Bacillaceae and *Saccharimonas* increased. *Bacillus* belongs to the Firmicutes phylum and is widely distributed in the oral mucosa, dental plaque, and other areas. It can produce bacteriocins (such as Subtilin), lipopeptides, and lysozyme, which inhibit the growth of oral pathogens (such as *Streptococcus mutans*), reduce the risk of dental caries and periodontitis, and cooperate with other bacteria to regulate biofilm formation, inhibit fungal overgrowth, and protect oral mucosal integrity (Aysha Jebin and Suresh, 2023). Further research is needed to determine whether its protective effect plays a role in PD. The correlation analysis between the abundance of the genus Saccharomyces and clinical symptoms showed a significant correlation with the decrease of NMSQ and FSS scores, indicating that the enrichment of Saccharomyces can alleviate non motor symptoms and fatigue in PD patients. However, its metabolic characteristics and functions are not fully understood, and further research such as metagenomic sequencing and metabolomics is needed to confirm.

Furthermore, we used PICRUSt to predict and analyze metabolic functions between the motor symmetric and asymmetric group. In the gut microbiota, compared with the symmetric group, the asymmetric group showed a predicted enrichment in pathways related to PI3K-Akt signaling, starch and sucrose metabolism, estrogen signaling pathway, acarbose and validamycin biosynthesis, antigen processing and presentation, and neomycin, kanamycin and gentamicin biosynthesis (Figure 4A). Among them, starch and sucrose metabolism are important pathways for bacteria to produce SCFA, which have a protective effect on PD (Zhao and Zhao, 2023). Epigenetic studies reveal that SCFA may protect the CNS through anti-inflammatory properties and reducing intestinal barrier leakage (Whittle and Singewald, 2014). Metabolic function prediction suggests that there are more active protective metabolic pathways in the intestine of the exercise asymmetric group, which is consistent with milder non exercise symptoms and supports the hypothesis that diseases in the exercise asymmetric group may not originate from the intestine. In the oral, compared with the asymmetric group, the symmetric group showed more abundant pathways related to the immune system, environment, digestion, pyrimidine metabolism, carbon fixation, niacin and nicotinamide metabolism, RNA degradation, and methane metabolism (Figure 4B). The activation of immune system pathways may drive neuroinflammatory responses and promote the occurrence and development of PD, but further research is needed to determine whether the mechanism underlying the promotion of symptom progression by oral microbiota is involved.

Several limitations should be acknowledged. First, the cross-sectional design restricts causal inference; thus, our work explores potential associations rather than establishing causality. Longitudinal studies are required to determine whether these microbial signatures are consequences or contributing factors. Second, the modest sample size increases the risk of false-positive findings in high-dimensional analyses. Consequently, our results should be interpreted as hypothesis-generating and require validation in larger cohorts. Third, although we minimized confounders by excluding active periodontal diseases and extreme diets, and confirmed that constipation severity and medication profiles were comparable between groups, residual confounding from unmeasured factors may persist. Finally, while strict aseptic protocols were implemented and the identified taxa aligned with canonical human microbiota rather than contaminants (Human Microbiome Project Consortium, 2012; Salter et al., 2014), the absence of sequenced negative controls prevents us from entirely ruling out background DNA in low-biomass samples. Future studies utilizing extraction blanks and multi-omics approaches are warranted.

In summary, among the two groups of PD patients with motor symmetric/asymmetric patterns, the symmetric group had higher H-Y staging, more severe daily life motor symptoms, and more severe salivation symptoms compared to the asymmetric group, but exhibited better cognitive function. There are structural differences in the gut and oral microbiota between the two groups of PD patients, with a more significant difference in gut microbiota diversity. Furthermore, predictive functional analysis suggested divergent metabolic potentials between the symmetric and asymmetric groups. Our findings reveal distinct gut microbiota profiles associated with motor symmetric/asymmetric patterns in PD, which aligns with the hypothesis that different disease origins may underlie these clinical subtypes. Our study describes the potential pathological interactions between microbiota and symmetric/asymmetric PD, providing valuable clinical data for further mechanism exploration.

## Data Availability

The datasets presented in this study can be found in online repositories. The names of the repository/repositories and accession number(s) can be found at: https://www.ncbi.nlm.nih.gov/, PRJNA1394324.
